# Risk of Secondary Pelvic Cancers Following Radiotherapy for Prostate Cancer

**DOI:** 10.3390/cancers18152382

**Published:** 2026-07-24

**Authors:** Mutlay Sayan, Yetkin Tuac, Crystal Hernandez, Yun Rose Li, Pamela W. Coleman, Mack Roach

**Affiliations:** 1 Department of Radiation Oncology, Brigham and Women’s Hospital, Dana-Farber Cancer Institute, Harvard Medical School, Boston, MA 02115, USA; 2 Department of Statistics, Ankara University, Ankara 06100, Türkiye; 3 Department of Radiation Oncology, University of California San Francisco, San Francisco, CA 94143, USA; 4 Department of Radiation Oncology, City of Hope Cancer Center, Duarte, CA 91010, USA; 5 Division of Urology, Howard University College of Medicine, Washington, DC 20059, USA

**Keywords:** prostate cancer, radiotherapy, secondary cancers, bladder cancer, rectal cancer, survivorship

## Abstract

**Background/Objectives**: Secondary pelvic cancer (SC) risk after radiotherapy (RT) for prostate cancer (PC) remains uncertain; we performed an updated synthesis of observational studies and randomized trials. **Methods**: We reviewed systematic reviews, meta-analyses, and randomized trials published in 2001–2025 evaluating SC risk after RT for PC. Given potential detection bias from RT-related rectal or bladder bleeding, the primary outcome was relative risk (RR) of death. **Results**: Across 12 reports—eight systematic reviews/meta-analyses and four randomized trials—comparators were predominantly RP or mixed cohorts; review-level sample sizes were not summed because of potential overlap. In randomized data with >10 years of follow-up, SC occurred in 25.3% of RT-treated patients versus 20.8% in RP/hormone therapy groups, while deaths from other cancers occurred in 8.5% versus 7.2%. The median converted RR of death from SC was 1.05. These estimates may be influenced by unmeasured lifestyle factors such as smoking and baseline comorbidities, which were not accounted for in observational cohorts and are more common among patients receiving RT, who are often less likely to be RP candidates. **Conclusions**: RT is associated with a small but consistent increase in SC, with a 4.5% absolute excess incidence over >10 years. The observed 1.3% absolute difference in deaths from other cancers was modest and similar in magnitude to the reported perioperative mortality range of approximately 0.2–1.1% after RP, although these risks occur over substantially different time horizons. The smaller excess seen in randomized trials than in retrospective studies suggests that much of the apparent risk in prior reports may reflect confounding and/or detection bias rather than direct radiation effect.

## Simple Summary

Radiotherapy is a common and effective treatment for prostate cancer, but patients often ask whether it can increase the risk of developing another cancer years later, especially in nearby organs such as the bladder, rectum, or colon. Prior studies have reported different estimates, partly because patients treated with surgery may be healthier than those treated with radiotherapy, making comparisons difficult. In this study, we reviewed published evidence from systematic reviews, meta-analyses, and randomized trials to better understand the true size of this risk. We found that radiotherapy may be associated with a small increase in second pelvic cancers, but the increase in deaths from these cancers appears modest. These findings may help clinicians discuss long-term risks more clearly with patients when choosing prostate cancer treatment and highlight the need for future studies that better account for baseline health, lifestyle factors, and modern radiotherapy techniques.

## 1. Introduction

Radiation oncologists are frequently asked to counsel patients about the long-term risk of second pelvic cancers (SCs)—including malignancies of the bladder, rectum, and colon—after definitive radiotherapy for localized prostate cancer. Although several population-based cohort studies and a limited number of randomized trials have attempted to quantify this risk [1,2,3,4], interpretation remains challenging due to substantial methodological limitations.

A major source of bias arises from the choice of control group. Many studies compare patients receiving radiotherapy to those treated with radical prostatectomy (RP)—a population with different baseline health profiles and survivorship patterns compared with either the general population or men receiving radiation.

Patients undergoing RP have consistently demonstrated significantly lower standardized mortality ratios (SMRs) for multiple malignancies, likely reflecting both favorable baseline health and selection bias. In a large single-institution series of over 18,000 patients, the SMR was 0.43 (95% CI, 0.29–0.57) for cancers of the colon, rectum, and anus, and 0.47 (95% CI, 0.22–0.73) for bladder cancer, relative to expected rates in the general population [5]. Such differences can inflate relative risk estimates for radiation-associated SCs when RP is used as the reference group. Further complicating interpretation is the heterogeneity in effect measure reporting across studies—including odds ratios, hazard ratios, standardized incidence ratios, and relative risks—which hinders direct comparison.

In this study, we re-examine the evidence on radiation-associated secondary cancers through an updated synthesis of systematic reviews, meta-analyses, and randomized trials. By harmonizing effect size metrics across heterogeneous sources, we sought to define realistic estimates of secondary cancer risk with the goal of improving clinical counseling, aligning patient expectations, and informing future survivorship research.

## 2. Materials and Methods

### 2.1. Evidence Acquisition

We conducted a comprehensive literature search to identify systematic reviews, meta-analyses, and randomized trials evaluating the risk of secondary cancers following radiotherapy for localized prostate cancer. Searches were performed in PubMed/MEDLINE for articles published between 2001 and 2025 using combinations of the terms: “prostate cancer”, “second cancer”, and “radiotherapy”. The final literature search was conducted on 2 January 2026. All identified records were screened by title and abstract. Full-text review was then performed for reports that met the initial screening criteria. Titles and abstracts and subsequently eligible full-text reports were screened by one reviewer, with eligibility decisions verified by a second reviewer. Disagreements were resolved through discussion.

**Figure 1:**
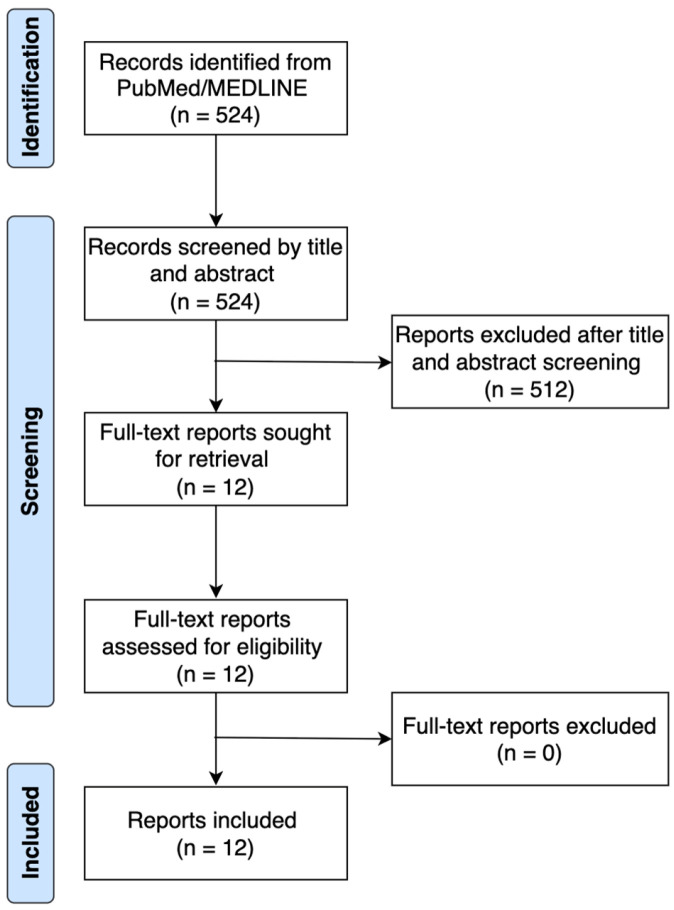
Preferred Reporting Items for Systematic Reviews and Meta-Analyses (PRISMA) flow diagram showing record identification, title and abstract screening, full-text eligibility assessment, and final inclusion of twelve studies (eight systematic reviews/meta-analyses and four randomized trials).

Eligibility criteria were prespecified using the Population, Intervention, Comparator, Outcomes, and Study Design (PICOS) framework and are summarized in Table 1. We excluded case reports, single-institution cohort studies, narrative reviews, and dosimetric modeling studies without clinical endpoints. Study selection and reporting followed Preferred Reporting Items for Systematic Reviews and Meta-Analyses (PRISMA) guidelines. The complete screening process, including the numbers of records identified, screened, excluded after title and abstract review, assessed in full text, and included in the final synthesis, is shown in Figure 1. A total of 524 records were screened by title and abstract, 512 were excluded at this stage, and 12 full-text reports were assessed for eligibility and included in the final synthesis. All identified records were screened by title and abstract. Full-text review was then performed for reports that met the initial screening criteria. This review was not registered, and a formal review protocol was not prepared.

### 2.2. Outcome Measures and Data Extraction

Radiotherapy can cause bleeding from the rectum or the bladder, resulting in the incidental diagnosis of a superficial non-life-threatening neoplasm; thus, the primary outcome of our analysis is relative risk estimates for deaths from SCs—specifically bladder, rectal, and colorectal cancers- while the incidence of SCs was a secondary endpoint. For each eligible study, we recorded the number of patients included, the type of comparator group (e.g., RP or general population [GP]), and the reported effect estimates, which could include standardized incidence ratios (SIR), odds ratios (OR), hazard ratios (HR), or relative risks (RR). When available, we also documented absolute or cumulative incidence estimates, the length of follow-up, and any subgroup analyses. Long-term follow-up was defined as at least ten years. Studies were grouped according to study design and outcome. Systematic reviews and meta-analyses were synthesized separately from randomized trials, and randomized trials reporting second-cancer incidence were presented separately from those reporting deaths from other cancers.

### 2.3. Statistical Analysis

Reported effect measures (ORs, HRs, and SIRs) were harmonized to approximate RRs to enable direct comparison across heterogeneous studies. ORs were converted to RRs using a validated method [6,7]:

$$PR=\frac{OR}{\left(1-P_{0}\right)+(P_{0}\cdot OR)}$$

where *P*_0_ represents the baseline incidence rates estimated from large population-based datasets [8,9].

SIRs and HRs were interpreted as RR approximations under the rare-outcome assumption and with long-term follow-up, consistent with prior methodological precedent. Studies that reported absolute risk without a comparator group were retained in the synthesis to contextualize long-term risks but were not included in RR estimation.

Randomized trials were evaluated separately from systematic reviews and meta-analyses. Study-specific crude risk ratios (RRs) and 95% confidence intervals (CIs) were calculated from the reported event counts. Because the trials differed in their outcome definitions, treatment settings, and comparator groups, a single pooled estimate across all randomized trials was not calculated.

## 3. Results

### 3.1. Study Selection and Characteristics

A total of 8 systematic reviews and meta-analyses (Table 2) [10,11,12,13,14,15,16,17] and 4 randomized trials (Table 3 and Table 4) [4,18,19,20,21] were identified and included for this analysis. Comparator groups varied across studies and included patients treated with RP, men from the GP, or were not specified (NS; often mixed prostate cancer cohorts managed with RP, active surveillance [AS]/watchful waiting [WW], or hormone therapy [HT]). When weighted by sample size, 26.6% of comparators were RP, 5.1% were GP, and 68.3% were NS. Within randomized trials, additional comparators included HT (43.9%) and active monitoring (AM) (17.2%), although these constituted <0.05% of the overall cohort. More than 95% of comparators across all studies were RP or NS cohorts, indicating that most available estimates of secondary cancer risk are driven by comparisons with surgically treated or RP-heavy control groups.

Event rates were calculated using the denominators reported in the original publications. Crude RRs and 95% CIs were calculated from the published event counts and do not account for censoring or time to event. The reported time-to-event HR was therefore retained for the overall SPCG-7 result. SPCG-7 additionally reported an increased risk of urinary bladder cancer with ET plus RT compared with ET alone (HR 2.54; 95% CI, 1.14–5.69). The three ProtecT comparisons share treatment groups and are not statistically independent; therefore, they were not combined in a pooled estimate.

### 3.2. Secondary Cancer Incidence and Deaths from Other Cancers

The incidence of secondary cancers was 25.3% (236 of 931) in patients who received RT versus 20.8% (194 of 934) in those who underwent RP or received HT, with median follow-up exceeding 10 years [18,19]. In trials where outcomes were reported as deaths from other cancers, pooled results showed 8.5% (98 of 1148) of RT patients and 7.2% (83 of 1155) of patients who underwent RP or received HT died from other cancers [20,21]. Among other comparators, 10.6% (58 of 545) of patients assigned to active monitoring died from other cancers. The median converted RR of death from SC was 1.05.

### 3.3. Bladder Cancer

Across all studies, RT was consistently associated with an increased risk of secondary bladder cancer. The median converted RR was 1.830 (range, 1.63–2.54), with no study reporting a null or protective association.

### 3.4. Rectal and Colorectal Cancers

Compared to bladder cancer, rectal and colorectal malignancies demonstrated slightly lower but still elevated risk estimates following RT. The median converted RR was 1.46 (range, 0.81–1.79). Most systematic reviews demonstrated modest increases, although estimates varied depending on comparator group and cancer site.

### 3.5. Randomized Trial Evidence

The randomized trials were evaluated separately because they provide comparisons that are less susceptible to treatment-selection bias and include clearly defined treatment groups. Trials reporting second-cancer incidence are summarized in Table 3, and trials reporting deaths from other cancers are summarized in Table 4. In the ProtecT trial, deaths from other cancers occurred in 58 of 545 patients assigned to active monitoring, 52 of 553 assigned to RP, and 54 of 545 assigned to RT. The crude RR was 0.88 (95% CI, 0.62–1.26) for RP versus active monitoring, 1.05 (95% CI, 0.73–1.51) for RT versus RP, and 0.93 (95% CI, 0.66–1.32) for RT versus active monitoring. Thus, none of the pairwise comparisons demonstrated a statistically clear difference in deaths from other cancers.

For second-cancer incidence, the crude RR was 1.35 (95% CI, 1.12–1.63) for RT plus endocrine therapy versus endocrine therapy alone in SPCG-7 and 0.99 (95% CI, 0.72–1.35) for immediate postoperative RT versus observation after RP in EORTC 22911. For deaths from other cancers, the crude RR was 1.42 (95% CI, 0.91–2.21) for ADT plus RT versus ADT alone in NCIC CTG PR3/MRC PR07. These estimates were not pooled because the trials evaluated different treatment settings, comparators, and outcome definitions.

## 4. Discussion

In this study, we found that patients treated with RT had an absolute increase of 4.5% in the incidence of secondary cancers and 1.5% in deaths from secondary cancers compared with those treated with RP or HT in randomized controlled trials. After harmonizing effect size metrics across heterogeneous studies, the median converted RR of death from SC was 1.05. Although these risks are modest, their consistency across study types highlights the importance of incorporating secondary cancer risk into survivorship counseling, particularly given the growing population of long-term prostate cancer survivors. These findings indicate that the absolute excess incidence and mortality associated with RT are small in magnitude. When contrasted with prior meta-analyses and retrospective studies—where relative risks were often substantially higher—the present synthesis suggests that much of the apparent excess risk may reflect residual confounding and differences in patient selection rather than a direct causal effect of radiation.

The present synthesis extends the existing literature by distinguishing incidence-based estimates from mortality-based outcomes and by evaluating randomized evidence separately from observational reviews. Most prior systematic reviews and meta-analyses focused on the risk of being diagnosed with a secondary cancer, with converted RRs generally ranging from approximately 1.46 to 1.83 for bladder, rectal, and colorectal cancers. In contrast, relatively few studies reported cancer-related mortality, and randomized trials generally reported deaths from all other cancers rather than deaths specifically attributable to secondary pelvic malignancies. The median converted RR for deaths from other cancers was 1.05, suggesting that the observed mortality difference was substantially smaller than incidence-based estimates might imply. This distinction is clinically important because incidence estimates may be influenced by surveillance and detection bias, including the diagnosis of superficial or nonlethal malignancies, whereas mortality-based outcomes may be more relevant to long-term survivorship counseling. However, interpretation remains challenging because the available studies differ in comparator groups, outcome definitions, latency periods, radiotherapy techniques, and adjustment for baseline health and lifestyle factors.

A critical challenge in quantifying the risk of radiation-associated second malignancies lies in the methodological variation across studies. Differences in comparator cohorts, latency thresholds, and confounder adjustment significantly impact risk estimates. In addition, RT-related rectal or bladder bleeding may prompt diagnostic workup and incidental detection of small polyps or superficial bladder cancers, introducing observation bias and potentially inflating incidence-based estimates. The majority of the studies used patients treated with RP as the referent group—an approach that may overstate relative risks due to selection bias and healthier baseline characteristics among surgically treated patients. For instance, a population-based study demonstrated that men undergoing RP had a significantly reduced SMR for subsequent cancers compared to the general population, including an SMR of 0.43 (95% CI, 0.29–0.57) for colorectal cancers and 0.47 (95% CI, 0.22–0.73) for bladder cancer [5]. These findings highlight how comparator choice can shape the interpretation of radiation-associated secondary cancer risk. Importantly, when placed in the context of other treatment-related risks, the observed 1.3% absolute difference in deaths from other cancers after RT over more than a decade of follow-up was similar in magnitude to the reported perioperative mortality range of approximately 0.2–1.1% after RP [22,23,24,25,26,27,28,29,30]. However, this comparison should be interpreted cautiously because perioperative mortality occurs shortly after surgery, whereas deaths from other cancers accumulate over long-term follow-up and cannot necessarily be causally attributed to RT. Moreover, in randomized trials, any small increase in SC mortality with RT should be weighed against its overall survival benefit, as improved prostate cancer outcomes may offset this risk. In the postoperative recurrence setting, RT also remains the only potentially curative local option for patients who recur after RP. Finally, variation in effect measures further complicates interpretation, as studies reported outcomes using SIRs, ORs, HRs, or RRs. To facilitate direct comparison, we converted estimates to approximate RRs, recognizing that this approach introduces assumptions—particularly when converting ORs in rare event settings—but improves interpretability and aligns with precedents in the literature.

The long latency of radiation-associated malignancies also complicates interpretation of the available evidence. Secondary solid cancers generally require many years to become clinically apparent, and studies with limited follow-up may therefore underestimate the true long-term risk. Conversely, studies with prolonged follow-up often include patients treated with older radiotherapy techniques, larger treatment fields, and less conformal dose distributions that may not reflect contemporary practice. Improvements in treatment planning, image guidance, and normal-tissue sparing have reduced radiation exposure to adjacent pelvic organs; however, whether these advances translate into a lower incidence of secondary malignancies remains uncertain because sufficiently mature long-term data are limited. The balance between treatment conformity and exposure to low doses of radiation outside the target volume may also differ across external-beam techniques and brachytherapy. Consequently, risk estimates derived from historical cohorts should not be directly applied to patients receiving modern radiotherapy without considering treatment era, irradiated volume, delivered dose, and expected duration of survival. Future studies should therefore report radiotherapy technique and dose distribution in greater detail and include sufficiently long follow-up to distinguish treatment-related malignancies from cancers arising because of aging, shared risk factors, or intensified surveillance.

An additional consideration is the role of lifestyle factors and treatment selection in shaping observed risks. Smoking, obesity, alcohol use, and baseline comorbidities such as cardiovascular or metabolic disease are well-established risk factors for bladder and colorectal cancers [31,32,33,34]. These exposures were rarely accounted for in observational cohorts and are often more prevalent among patients receiving RT, who are generally older and less likely to be surgical candidates for these very reasons. As a result, the higher relative risks reported in registry-based studies likely reflect confounding by baseline health status and lifestyle, rather than a direct causal effect of radiation. This interpretation is supported by data from randomized trials, where such factors should be more evenly distributed across treatment groups, and where the absolute excess risks associated with RT were smaller than those observed in population-based studies.

The ProtecT trial provides a particularly informative comparison because patients were randomized among active monitoring, RP, and RT and followed for 15 years. Deaths from other cancers were similar across all three groups, including RP versus active monitoring (crude RR, 0.88; 95% CI, 0.62–1.26). Thus, within a randomized population, RP was not associated with clearly lower other-cancer mortality than a noninterventional management strategy. This contrasts with retrospective studies in which patients undergoing RP often appear to have substantially lower risks of subsequent malignancy or non–prostate cancer mortality, supporting the possibility that favorable baseline health and treatment-selection factors contribute to those observational associations. Nevertheless, ProtecT reported deaths from all other cancers rather than deaths specifically attributable to secondary pelvic malignancies, and therefore provides reassurance regarding overall other-cancer mortality but cannot isolate radiation-associated pelvic cancer mortality.

Advances in molecular profiling and artificial intelligence (AI)–driven platforms offer new opportunities to better identify patients at risk for radiation-associated second cancers. The Decipher genomic classifier, originally developed to predict metastasis and prostate cancer–specific mortality, has more recently been applied to broader prognostic modeling across oncology [35,36,37]. Similarly, multimodal AI models that integrate clinical features with digitized histopathology have shown potential to improve individualized risk prediction and support more precise clinical decision-making [38,39]. In addition, germline biomarkers, including single nucleotide polymorphisms (SNPs) linked to radiation sensitivity and DNA repair capacity, may further refine patient selection [40,41,42]. Incorporating these tools into prospective cohorts with long-term follow-up will be essential to translate these biological advances into personalized counseling and risk-reduction strategies.

Socioeconomic position and access to health care may represent additional sources of residual confounding. In some health-care systems, patients undergoing RP may be more affluent, have private insurance, and have greater access to specialist and preventive care. These factors may also be associated with healthier lifestyles, earlier cancer detection, lower comorbidity, and longer life expectancy. Consequently, the higher cancer risk observed after RT in comparisons with RP-treated populations may partly reflect differences in socioeconomic status, baseline health, and access to care rather than a direct effect of RT; such estimates may therefore be better interpreted as an upper bound of the excess risk associated with RT rather than as an average causal effect.

Although this study has several strengths—including a comprehensive synthesis across multiple study types and the use of harmonized effect estimates—it also has limitations. First, formal meta-analysis was not feasible due to heterogeneity in study design, reported outcomes, and population characteristics. In addition, the included systematic reviews and meta-analyses likely shared primary studies and underlying registry populations, and the extent of this overlap could not be reliably determined. Second, adjustment for important confounders such as smoking and baseline comorbidities was rarely performed, making it difficult to determine whether the observed associations reflect a true effect of radiation or differences in underlying patient risk. Third, this review relied on published literature, raising the possibility of publication bias and overestimation of effect sizes. Formal assessment of publication bias using funnel plots was not performed because the available estimates were overlapping, heterogeneous, and insufficient in number within any clinically comparable outcome group. Fourth, formal study-level risk-of-bias and certainty-of-evidence assessments were not performed; therefore, differences in methodological quality across the included reports were considered narratively rather than incorporated into the synthesis. These limitations highlight the need for large-scale, prospectively designed studies with standardized reporting and long-term follow-up to more accurately define secondary cancer risk after prostate RT.

## 5. Conclusions

Pelvic radiation for prostate cancer is associated with a small but consistent increase in the risk of being diagnosed with secondary bladder and rectal cancers, with an even smaller increase in the risk of death from SCs. Although the absolute risks are low, they are clinically meaningful in the setting of improving prostate cancer survival and should be incorporated into shared decision-making and survivorship care. Looking forward, integration of genomic, AI-based, and clinical risk predictors into prospective cohorts with long-term follow-up will be essential to refine counseling and support personalized risk-reduction strategies.

## Figures and Tables

**Table 1:** PICOS framework and inclusion criteria.

PICOS Component	Inclusion Criterion
Population	Patients with localized prostate cancer
Intervention	External beam radiotherapy for prostate cancer
Comparator	Radical prostatectomy, general population, hormone therapy, active surveillance, not specified
Outcomes	Incidence of second pelvic malignancies (bladder, rectal, colorectal cancers)
Study Design	Systematic reviews, meta-analyses, and randomized trials

**Table 2:** Summary of Systematic Reviews and Meta-Analyses Evaluating Second Pelvic Cancers After Radiotherapy for Prostate Cancer.

Author (Year)	No. of Studies	No. of Patients	Control Group *	Outcome and Reported Effect Estimate (95% CI) **	Converted RR	Conclusion
Murray (2014) [10]	47	1,047,492	RP (37.9%) GP (28.6%) NS (33.5%)	Rectal cancer: OR 1.60 (1.29–1.99) Bladder cancer: OR 1.63 (1.44–1.84)	1.60 (rectal cancer) 1.63 (bladder cancer)	The available evidence suggested a small increase in secondary bladder and rectal cancers after prostate RT, particularly with longer follow-up.
Jin (2014) [11]	4	647,857	RP (1.7%) NS (98.3%)	Colon cancer: SIR 1.47 (0.77–2.78) Rectal cancer: SIR 1.25 (0.85–1.84) Bladder cancer: SIR 1.69 (1.02–2.81)	1.47 (colon cancer) 1.25 (rectal cancer) 1.69 (bladder cancer)	RT was associated with a modest increase in secondary malignancy risk, which became apparent after more than 10 years of follow-up.
Lee (2016) [13]	16	33,231	RP (26.0%) GP (10.2%) NS (63.8%)	Rectal cancer: SIR 1.08 (0.68–1.72)	1.08 (rectal cancer)	Overall prostate RT was not associated with a statistically clear increase in secondary rectal cancer, although an increased risk was observed in the external beam RT subgroup.
Wallis (2016) [12]	21	930,843	RP (31.7%) NS (68.3%)	Bladder cancer: HR 1.67 (1.55–1.80) Rectal cancer: HR 1.79 (1.34–2.38) Colon cancer: HR 1.79 (1.34–2.38)	1.67 (bladder cancer) 1.79 (rectal cancer) 1.79 (colorectal cancer)	RT was associated with increased risks of secondary bladder, rectal, and colorectal cancers, although the reported absolute risks were low.
Rombouts (2018) [15]	23	719,823	RP (11.8%) NS (88.2%)	Rectal cancer: RR 1.36 (1.10–1.67)	1.36 (rectal cancer)	Prostate RT was associated with a modest increase in secondary rectal cancer risk, although the findings did not support changes to rectal cancer surveillance guidelines.
Zhu (2018) [14]	16	1,216,687	RP (11.6%) NS (88.4%)	Rectal cancer: OR 1.64 (1.39–1.94) Colon cancer: OR 1.33 (1.02–1.76)	1.63 (rectal cancer) 1.32 (colon cancer)	RT was associated with an increased risk of secondary rectal cancer; the association with colon cancer was less consistent across effect measures.
Nugent (2022) [16]	8	796,386	RP (9.2%) NS (90.8%)	Rectal cancer: OR 1.45 (1.07–1.97)	1.44 (rectal cancer)	Prostate RT was associated with increased odds of subsequent rectal cancer, although the absolute risk remained small.
Matsukawa (2025) [17]	31	576,341	RP (100%)	Bladder cancer: OR 2.23 (1.92–2.58) at 5 yrs OR 2.18 (1.98–2.41) at 10 yrs OR 1.91 (1.76–2.07) at 15 yrs OR 1.77 (1.65–1.90) at 20 yrs	2.22 at 5 yrs 2.17 at 10 yrs 1.90 at 15 yrs 1.76 at 20 yrs	RT was associated with an increased risk of secondary bladder cancer compared with RP at all evaluated follow-up intervals.

Abbreviations: RP = Radical Prostatectomy; GP = General Population; NS = Not Specified; SIR = Standardized Incidence Ratio; HR = Hazard Ratio; OR = Odds Ratio; RR = Relative Risk; yrs = years. * The “Not Specified” category may represent prostate cancer patients managed with radical prostatectomy, active surveillance/watchful waiting, or hormone therapy alone. ** Reported effect estimates are presented as published in the original reviews. Converted RRs were calculated to facilitate comparison across studies. Review-level estimates were not pooled across publications because of potential overlap in primary studies and underlying patient populations; consequently, the reported patient numbers should not be summed.

**Table 3:** Randomized Trials Reporting Second-Cancer Incidence After Prostate Cancer Treatment.

Trial	Randomized Comparison	No. of Patients	Follow-Up	Outcome	Events in Group 1	Events in Group 2	Effect Estimate (95% CI)	Interpretation
SPCG-7 [18]	ADT + RT vs. ADT alone	860	12.2 yrs	Any second cancer	168/429 (39.2%)	125/431 (29.0%)	RR: 1.19 (0.92–1.54)	Overall second-cancer incidence was higher in the RT group, and bladder cancer risk was also increased.
EORTC 22911 [19]	Immediate post-RP RT vs. observation	1005	10.6 yrs	Any second cancer	68/502 (13.5%)	69/503 (13.7%)	RR: 0.99 (0.72–1.35)	No statistically clear difference in second-cancer incidence.

Abbreviations: ADT, androgen-deprivation therapy; CI, confidence interval; RP, radical prostatectomy; RR, risk ratio; RT, radiotherapy; yrs, years.

**Table 4:** Randomized Trials Reporting Deaths from Other Cancers After Prostate Cancer Treatment.

Trial	Randomized Comparison	No. of Patients	Follow-Up	Outcome	Events in Group 1	Events in Group 2	Effect Estimate (95% CI)	Interpretation
NCIC CTG PR3/MRC PR07 [20]	ADT + RT vs. ADT alone	1205	8.0 yrs	Death from other cancers	44/603 (7.3%)	31/602 (5.1%)	RR: 1.42 (0.91–2.21)	No statistically clear difference.
ProtecT [21]	RT vs. RP	1643	15 yrs	Death from other cancers	54/545 (9.9%)	52/553 (9.4%)	RR: 1.05 (0.73–1.51)	No statistically clear difference.
	RT vs. AM				54/545 (9.9%)	58/545 (10.6%)	RR: 0.93 (0.66–1.32)	No statistically clear difference.
	RP vs. AM				52/553 (9.4%)	58/545 (10.6%)	RR: 0.88 (0.62–1.26)	No statistically clear difference.

Abbreviations: ADT, androgen-deprivation therapy; AM, active monitoring; CI, confidence interval; RP, radical prostatectomy; RR, risk ratio; RT, radiotherapy; yrs, years.

## Data Availability

The original contributions presented in this study are included in the article. Further inquiries can be directed to the corresponding author.

## Abbreviations

The following abbreviations are used in this manuscript:

AI	Artificial intelligence
AM	Active monitoring
AS	Active surveillance
CI	Confidence interval
EORTC	European Organisation for Research and Treatment of Cancer
GP	General population
HR	Hazard ratio
HT	Hormone therapy
OR	Odds ratio
PC	Prostate cancer
PICOS	Population, Intervention, Comparator, Outcomes, and Study Design
PRISMA	Preferred Reporting Items for Systematic Reviews and Meta-Analyses
RP	Radical prostatectomy
RR	Relative risk
RT	Radiotherapy
SC	Secondary cancer
SIR	Standardized incidence ratio
SMR	Standardized mortality ratio
SNP	Single nucleotide polymorphism
SPCG-7	Scandinavian Prostate Cancer Group-7
WW	Watchful waiting
